# XIS-humidity: a daily spatiotemporal machine-learning model for dew point in the contiguous United States

**DOI:** 10.1088/2515-7620/ae5c6c

**Published:** 2026-04-15

**Authors:** Kodi B Arfer, Allan C Just

**Affiliations:** Center for Epidemiologic Research, School of Public Health, Brown University, Providence, RI 02903, United States of America

**Keywords:** machine learning, humidity, dew point, heat index, remote sensing, land-use regression

## Abstract

Health effects of humidity are theoretically important but have appeared inconsistently in epidemiological studies. Better measures of humidity may help. We construct a machine-learning model of dew point covering the US from 2003 through 2024, using the same XGBoost-IDW synthesis (XIS) framework as our previously published models of temperature and particulate matter. XIS-Humidity predicts three daily variables at high spatial resolution: mean humidity; T-min humidity (the humidity observed when temperature is at its minimum); and T-max humidity (similarly, for maximum temperature). In 2024, we obtain RMSEs of 1.30 K for mean humidity, 1.72 K for T-min, and 2.07 K for T-max, compared to SDs above 10 K. For mean humidity, as well as a metric of heat index computed with the help of XIS-Temperature, XIS-Humidity is more accurate than two competing models (NLDAS-2 and PRISM) in most years. We also find a greater association of social vulnerability with heat index when using XIS. Compared to previous models of humidity or heat index, XIS-Humidity should allow for more accurate and sophisticated analyses in the epidemiology of humidity and temperature.

## 1. Introduction

It has been generally acknowledged for some time that it’s not the heat; it’s the humidity [1]. Global warming has led to increases in humidity (as warmer air can hold more moisture) as well as situations of extreme humid heat, producing wet-bulb temperatures that exceed the limits of human physiology [2]. Low humidity on cold days may also be dangerous, by increasing the spread of respiratory viruses [3, 4].

Despite good theoretical reasons for humidity to worsen the effects of heat, many studies have failed to confirm a positive relationship of humidity with heat-related illness [5]. Partly, this may be due to how humidity is measured. Different humidity metrics are most appropriate for different research questions. Relative humidity is a common choice, but not always the best [3, 6]. For example, Shaman and Kohn’s [7] reanalysis of Lowen *et al*’s [8] study of influenza in guinea pigs found stronger effects of absolute humidity than relative humidity. Furthermore, relative humidity is by nature directly affected by temperature. One day may have air just as moist as another, but a higher relative humidity, because of a lower temperature. This inherent confounding of relative humidity with temperature is problematic for distinguishing health effects of humidity and heat.

A logical way to get humidity estimates, at the kind of spatiotemporal resolution needed for epidemiology, is to use satellite measures. The Moderate Resolution Imaging Spectroradiometer (MODIS) instrument on NASA’s Terra and Aqua satellites provides daily measures of surface temperature and column water vapor. Studies of limited temporal extent in Spain [6, 9], Malaysia [10], and China [11] have shown associations of MODIS variables with humidity variables, such as specific humidity and vapor pressure, measured from ground stations or balloons. These models succeed as proofs of concept but eschew typical land-use-regression predictors, such as elevation, that should be expected to improve accuracy. Another approach is represented by downscaling and interpolation models of atmosphere, such as the North American Land Data Assimilation System-2 (NLDAS-2) [12] and the Parameter-elevation Relationships on Independent Slopes Model (PRISM) [13]. NLDAS-2 and PRISM cover large swaths of spacetime but don’t synthesize additional predictors including MODIS.

In this paper, we construct a model of humidity using the XGBoost-IDW synthesis (XIS) framework that we previously used for temperature (XIS-Temperature) [14] and particulate matter [15]. The new model, XIS-Humidity, is particularly similar to XIS-Temperature, facilitating studies of temperature and humidity in combination. XIS uses MODIS data as well as a parsimonious set of additional predictors to obtain high predictive accuracy in a computationally modest package. The ability of XIS to make predictions for arbitrary points over land is ideal for epidemiological studies, and XIS-Humidity’s consideration of three daily humidity variables allows investigation of the sub-daily effects that may be important for health studies [5].

## 2. Method

### 2.1. Study area and time period

XIS-Humidity covers the same area and time period as XIS-PM_2.5_ and XIS-Temperature, namely the contiguous US (excluding large water bodies) for 2003 through 2024. Space is represented as floating-point longitude-and-latitude pairs, and time as days in midnight-to-midnight intervals of Central Standard Time (UTC−6).

### 2.2. Data

#### 2.2.1. Humidity

We represent humidity as the dew point, in kelvins. Dew point is the temperature to which air would have to be cooled to achieve a relative humidity of 100%, when water vapor would condense. Relative humidity is a more common metric, but as discussed above, its definition in terms of temperature is a weakness when one wishes to model temperature and humidity separately, or model their effects on health separately. We opt for dew point because it’s present in our data source more often than specific humidity (another mass-based humidity metric) and because we find the temperature units of dew point more intuitive than the mass-ratio units of specific humidity.

Analogously to XIS-Temperature, we represent the quantity of interest as three dependent variables (DVs): temperature-minimum (hereinafter ‘T-min’) humidity, mean humidity, and temperature-maximum (hereinafter ‘T-max’) humidity. Mean humidity is computed very similarly to mean temperature (using all observations in the day, weighted according to time), but for extrema, we don’t actually use the extreme observed humidity value on each day. Instead, we use the humidity observed when the temperature is at its own extremum: for example, T-max humidity is the dew point that’s observed when the same station reports the highest temperature of that day. The advantage of this approach is that when a health model uses both max temperature and T-max humidity, we can be assured that these variables represent genuinely co-occurring conditions. Also, informal checks suggest that the max daily heat index is better approximated when we compute heat index with T-max humidity than with the actually observed greatest humidity.

As in XIS-Temperature, we draw observations from the National Mesonet and COOP datasets in the Meteorological Assimilation Data Ingest System (MADIS) [16]. We only keep a dew-point observation if temperature is present as part of the same observation. We use the same cleaning and processing steps as for XIS-Temperature except that we omit the spatial-consistency check, because we feel less sure about how dew point can reasonably be expected to vary over space. We remove dew-point observations beyond NOAA’s record historical temperatures [17], since no equivalent exists for dew point.

#### 2.2.2. Predictors

**Figure 1.**
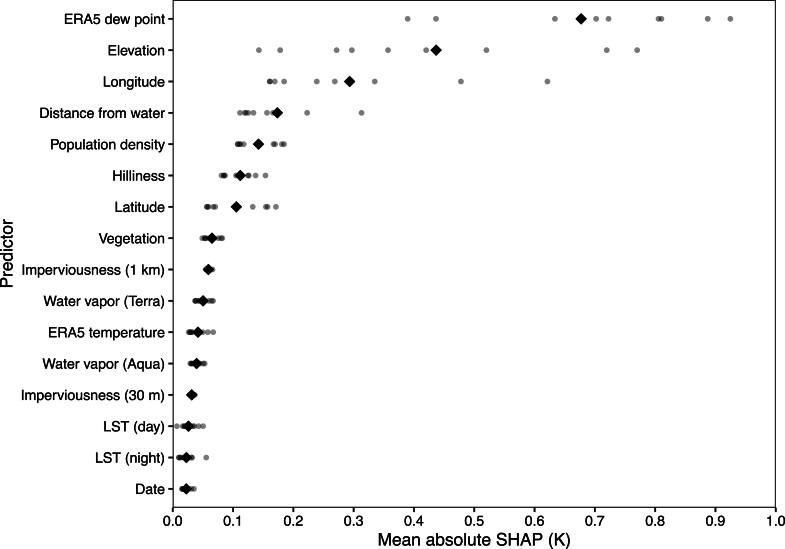
Mean absolute SHAP of each predictor in 2010 for mean humidity (the IDW feature, which has much greater absolute SHAP than everything else, is omitted). Small dots show per-region means. Diamonds show overall means.

XIS-Humidity uses all 13 predictors used by XIS-Temperature as well as the following, for a total of 17 (figure 1).

• Temperature and humidity (dew point) in kelvins, from the land component of the fifth generation of European reanalysis (ERA5-Land) [18]. These variables are available at 0.1° hourly resolution; we process the hourly variables to produce daily variables analogous to the corresponding DV, so XIS-Humidity uses T-max humidity from ERA5-Land when predicting T-max humidity. When ERA5-Land values are missing, which happens most often along coasts, we instead use the values from plain ERA5 [19], which provides these variables at a coarser spatial resolution of 0.125°.
• Column water vapor, in centimeters, from the MAIAC algorithm for Terra and Aqua (one variable per satellite) [20]. One value is given per day and 927-m grid cell. We use all non-missing values with quality bits 0 through 2 not equal to 0b011 (decimal 3), which would mean a cloudy pixel.

### 2.3. Modeling

The modeling and model-evaluation approach for XIS-Humidity is largely identical to that of our previous XIS models [14]. We model each year and DV separately. Inverse-distance weighting (IDW) supplies an estimate for each observation, and the difference between observations and IDW estimates is modeled with extreme gradient boosting (XGBoost, tuned per table 1). We evaluate the models with 5-fold station-level cross validation (CV). As for our previous models [14], we choose the hyperparameters by starting with 50 hyperparameter vectors randomly sampled with a maximin Latin-hypercube algorithm [21], evaluating each with CV with our data from 2004 and 2018, and making final manual selections on the basis of (a) computation time, (b) mean root mean square error (RMSE) across regions, and (c) max RMSE across regions.

**Table 1.** Selected hyperparameters for the three dependent variables. ‘Time’ is the sum of the time taken to cross-validate the model in the years used for tuning, 2004 and 2018. ‘RMSEZ’ reflects the weighted root mean square error from cross-validation, standardized into *z*-scores across all climate regions in both years; ‘RMSEZ (mean)’ is the mean of all such *z*-scores and ‘RMSEZ (max)’ is their maximum.

dv	nrounds	max_depth	eta	Gamma	Lambda	Alpha	Time (s)	RMSEZ (mean)	RMSEZ (max)
humid.min	500	9	0.078	0.38	360	0.57	894	−0.606	0.066
humid.mean	500	9	0.078	0.38	360	0.57	932	−0.615	−0.107
humid.max	500	9	0.051	0.37	120	0.28	983	−0.644	−0.004

## 3. Results

### 3.1. Cross-validation

Overall cross-validation results are shown in table 2. The RMSEs show large improvements over the SDs for all years and DVs. The RMSEs shrink further in later years, reaching around 1.75 K for T-min humidity, 1.3 K for mean, and 2 K for T-max. The number of stations per year is negatively Kendall-correlated with RMSE: −0.72 for T-min, −0.61 for mean, and −0.65 for T-max. The bias of our predictions per year range from −0.031 to +0.072 K for T-min, −0.023 to +0.029 K for mean, and −0.017 to +0.081 K for T-max. Tables 3 and 4 show that unweighted results at isolated stations and on hot station-days, respectively, are similar to the overall results. On the other hand, table 5 shows that RMSE can vary substantially by region. The mean-humidity model is most accurate in the Southeast and least accurate in the West.

**Table 2.** Weighted SDs and RMSEs (K) from yearly CV.

Year	Observations	Sites	T-min humid.		Mean humid.		T-max humid.	
			SD	RMSE	SD	RMSE	SD	RMSE
2003	648,350	3,532	11.11	2.39	10.67	1.88	10.56	2.74
2004	892,284	4,437	10.86	2.24	10.47	1.77	10.42	2.55
2005	1,341,232	6,377	10.90	2.20	10.47	1.69	10.36	2.44
2006	1,801,417	7,700	10.75	2.40	10.40	1.80	10.45	2.62
2007	2,124,024	8,762	11.38	2.29	10.94	1.75	10.89	2.69
2008	2,255,090	9,656	11.35	2.01	10.87	1.45	10.77	2.27
2009	2,450,442	9,762	11.22	1.99	10.80	1.46	10.75	2.22
2010	2,649,467	10,607	11.05	1.92	10.68	1.40	10.63	2.18
2011	2,963,815	11,751	11.23	1.95	10.79	1.41	10.80	2.24
2012	3,445,025	12,864	10.68	1.89	10.39	1.37	10.56	2.20
2013	3,665,525	13,856	11.52	1.86	11.10	1.37	11.12	2.13
2014	3,901,532	14,236	11.68	1.87	11.22	1.37	11.18	2.14
2015	4,025,494	14,782	11.07	1.76	10.67	1.28	10.73	2.05
2016	4,031,234	14,934	10.39	1.78	10.19	1.28	10.42	2.06
2017	4,222,205	14,795	10.87	1.75	10.60	1.26	10.74	2.02
2018	4,233,646	14,979	11.65	1.82	11.26	1.34	11.35	2.07
2019	4,744,673	19,231	11.47	1.75	11.02	1.28	11.02	2.05
2020	5,362,541	19,276	10.83	1.79	10.58	1.29	10.82	2.05
2021	5,806,989	20,613	11.18	1.78	10.95	1.29	11.20	2.06
2022	6,224,640	21,493	11.99	1.92	11.46	1.45	11.42	2.17
2023	6,500,398	22,668	10.67	1.74	10.34	1.28	10.45	2.04
2024	7,006,895	23,704	10.65	1.72	10.41	1.30	10.62	2.07

**Table 3.** Unweighted SDs and RMSEs (K) for isolated stations in yearly CV. A station is considered isolated if it’s at least 12 km away from all other stations on the same year. This is the same threshold we use for XIS-Temperature, but different stations may be selected, since we ingest MADIS separately for temperature and humidity.

Year	Observations	Sites	T-min humid.		Mean humid.		T-max humid.	
			SD	RMSE	SD	RMSE	SD	RMSE
2003	345,247	1,846	10.59	2.24	10.19	1.77	10.16	2.58
2004	459,557	2,256	10.37	2.10	10.07	1.64	10.08	2.39
2005	576,246	2,582	10.59	2.11	10.21	1.61	10.15	2.32
2006	719,974	2,864	10.39	2.27	10.09	1.72	10.15	2.50
2007	806,318	3,082	11.06	2.25	10.66	1.73	10.61	2.64
2008	808,590	3,232	11.05	1.90	10.56	1.35	10.46	2.14
2009	855,644	3,316	10.82	1.85	10.44	1.34	10.43	2.10
2010	876,054	3,359	10.71	1.80	10.39	1.32	10.38	2.06
2011	901,601	3,423	10.81	1.82	10.44	1.31	10.47	2.11
2012	984,479	3,584	10.36	1.80	10.10	1.30	10.24	2.12
2013	977,581	3,628	11.22	1.79	10.84	1.31	10.86	2.07
2014	1,018,404	3,630	11.40	1.79	10.96	1.30	10.91	2.07
2015	1,003,914	3,611	10.97	1.72	10.59	1.23	10.65	1.99
2016	1,015,493	3,702	10.10	1.69	9.94	1.20	10.17	1.99
2017	1,073,317	3,709	10.61	1.70	10.38	1.22	10.52	1.99
2018	1,080,013	3,736	11.41	1.75	11.07	1.28	11.18	2.01
2019	976,437	3,637	11.26	1.77	10.84	1.27	10.86	2.04
2020	1,059,706	3,757	10.61	1.77	10.38	1.27	10.62	2.03
2021	1,038,662	3,735	10.97	1.75	10.80	1.25	11.05	2.03
2022	1,054,246	3,692	11.86	1.82	11.37	1.35	11.31	2.09
2023	1,046,786	3,772	10.52	1.76	10.22	1.28	10.33	2.01
2024	1,088,311	3,816	10.59	1.75	10.37	1.31	10.55	2.05

**Table 4.** Unweighted SDs and RMSEs (K) from station-days with a max temperature of 92 °F (33.33 °C) or greater, taking the subset of station-days from XIS-Temperature that meet this criterion and are also included in XIS-Humidity.

Year	Observations	Sites	T-min humid.		Mean humid.		T-max humid.	
			SD	RMSE	SD	RMSE	SD	RMSE
2003	50,115	2,401	12.25	2.40	11.34	1.99	10.86	2.87
2004	47,151	2,545	12.66	2.27	11.93	1.80	11.48	2.74
2005	100,699	4,405	13.88	1.99	13.01	1.64	12.30	2.51
2006	149,143	5,881	13.30	2.36	12.31	1.79	11.66	2.72
2007	168,784	6,631	13.47	2.38	12.63	2.02	12.15	3.35
2008	138,102	6,404	13.61	1.88	12.50	1.49	11.73	2.41
2009	151,245	6,706	13.33	1.87	12.32	1.48	11.62	2.43
2010	208,505	7,870	14.61	1.60	13.97	1.34	13.54	2.24
2011	255,910	8,651	14.18	1.66	13.25	1.37	12.65	2.32
2012	295,915	10,160	12.61	1.72	11.84	1.40	11.52	2.38
2013	234,390	10,061	13.49	1.75	12.56	1.40	12.14	2.31
2014	243,929	9,186	13.20	1.81	12.31	1.44	11.93	2.38
2015	289,618	10,568	12.75	1.62	11.91	1.34	11.49	2.23
2016	317,984	11,310	12.82	1.62	12.32	1.31	12.26	2.19
2017	281,560	10,531	12.24	1.70	11.58	1.37	11.42	2.29
2018	323,029	11,092	13.35	1.66	12.68	1.38	12.45	2.26
2019	365,470	13,114	13.48	1.64	12.74	1.34	12.36	2.21
2020	458,582	15,335	11.82	1.80	11.25	1.39	11.22	2.25
2021	455,474	16,057	11.48	1.76	11.03	1.37	11.13	2.21
2022	555,159	17,545	12.80	1.74	12.00	1.36	11.69	2.15
2023	473,109	16,962	12.51	1.66	11.93	1.30	11.80	2.16
2024	629,407	18,878	11.84	1.66	11.35	1.33	11.33	2.18

**Table 5.** Weighted SDs and RMSEs (K) for 2010 broken down by NOAA climate region [22, 23].

Region	Observations	Sites	T-min humid.		Mean humid.		T-max humid.	
			SD	RMSE	SD	RMSE	SD	RMSE
Ohio Valley	260,288	1,013	11.21	1.37	10.83	1.05	10.68	1.77
Upper Midwest	231,240	890	12.22	1.48	11.56	1.07	11.10	1.72
Northeast	341,466	1,357	10.54	1.90	10.36	1.45	10.34	2.14
Northwest	345,797	1,377	6.77	1.95	6.40	1.43	6.65	2.19
South	249,432	963	10.95	1.71	10.51	1.22	10.61	2.06
Southeast	316,495	1,285	10.69	1.33	10.43	1.04	10.62	1.90
Southwest	336,663	1,356	8.97	2.43	8.13	1.68	7.93	2.52
West	393,390	1,629	8.11	2.48	8.00	1.80	8.64	2.59
Northern Rockies and Plains	174,696	737	10.60	2.07	9.79	1.60	9.25	2.34

#### 3.1.1. Model comparison

XIS is a fairly parsimonious model by comparison to many other statistical models of environmental variables. Still, it’s much more complex than, for example, IDW alone. To assess how well the model complexity of XIS contributes to predictive accuracy, we compare XIS for mean humidity in 2010 to two other methods: IDW alone, and linear regression by ordinary least squares (OLS) with the same predictors as in XIS. We find RMSEs of 1.73 K for IDW alone and 1.54 K for OLS, compared to 1.40 K for XIS. Thus is demonstrated the general principle of diminishing returns in model complexity, whereby large increases in complexity are necessary for relatively small improvements in accuracy [24, 25](see e.g. section 9 of [24], or chapter 7 of [25]).

#### 3.1.2. Feature contributions

Figure 1 shows a mean absolute SHapley Additive exPlanations (SHAP) [26] for each feature. SHAPs can be interpreted analogously to the terms of a linear-regression model: a SHAP of +2.5 for a given predictor and case means that the model attributes a +2.5 increase in its prediction for that case to that predictor. We see that the humidity variable from ERA5 is the most important predictor (other than IDW), which makes sense since it’s just another estimate of the DV. The effect for longitude reflects the greatly varying climates across the US, and elevation and the distance from water are logically related to the amount of moisture in the air.

**Figure 2.**
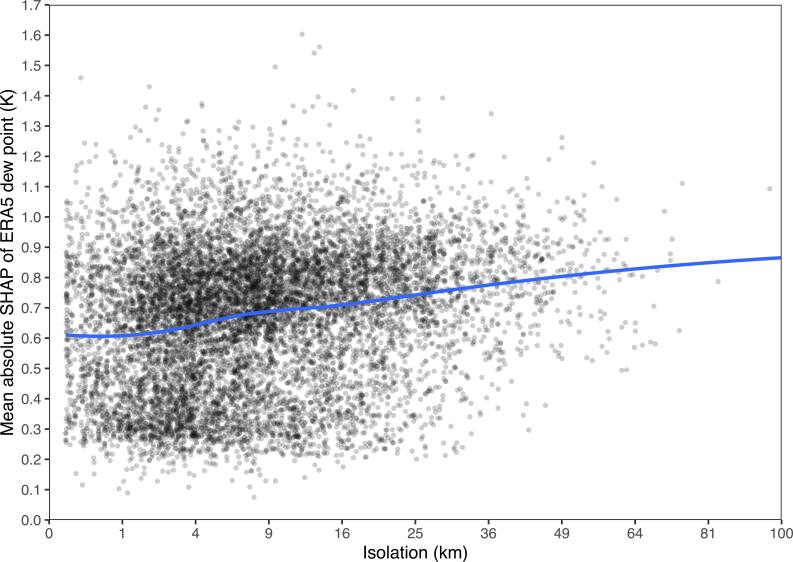
SHAP of ERA5 humidity as a function of site isolation. The *x*-axis is on a square-root scale.

**Figure 3.**
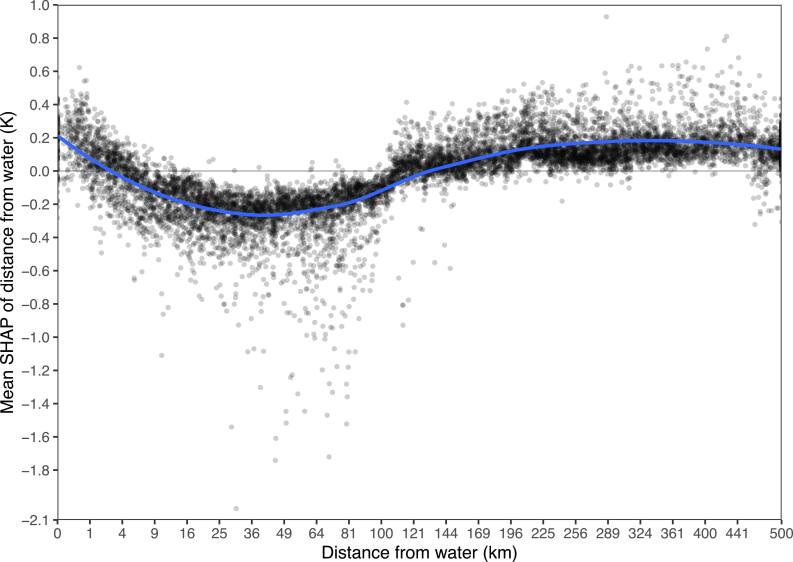
SHAP of distance from water as a function of distance from water. The *x*-axis is on a square-root scale.

**Figure 4.**
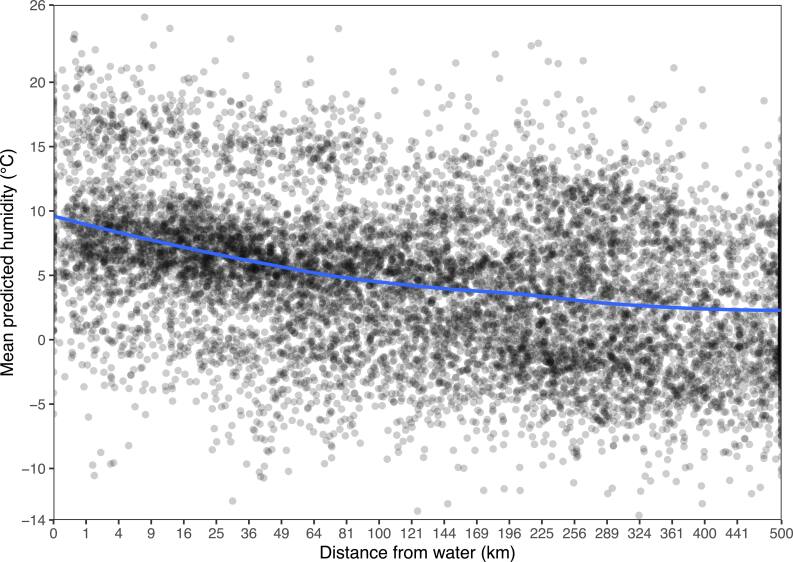
Humidity predictions as a function of distance from water. The *x*-axis is on a square-root scale.

Mean absolute SHAPs are simple but lack detail, and can’t clearly demonstrate nonlinear effects or interactions. Figures 2 and 3 depict the SHAPs of two predictions in greater detail, with trendlines via locally estimated scatterplot smoothing (LOESS). We see that XIS-Humidity makes greater use of ERA5 humidity as station isolation increases (and hence the utility of IDW decreases), while distance from water is most important at distances around 35 km. Figure 4 shows that the relationship between distance from water and the mean of XIS’s predictions of humidity (as opposed to the SHAP of distance from water alone) is still monotonic, as would be expected.

#### 3.1.3. Comparison with third-party models

We compare XIS’s accuracy in predicting MADIS dew point, as well as heat index, with two models developed by other groups:

• NLDAS-2 provides one value of temperature, air pressure, and specific humidity per hour and 0.125° grid square. From air pressure and specific humidity, we can compute dew point with the algorithm implemented by MetPy 1.6.2 [27].
• PRISM publicly provides one value for mean temperature and mean dew point per day and 2.5-arcminute grid square. (This resolution equals about 0.0417°, and is labeled by PRISM as ‘25m’ or ‘4km’. Higher resolutions, as well as full-year 2024 products, were publicly released too recently to be analyzed here.)

Two DVs are considered: mean daily dew point and daily heat index. We compute heat index from each model’s daily means of dew point and temperature (drawing on XIS-Temperature for temperature predictions, in the case of XIS) using the R package weathermetrics [28]. For XIS, predictions are taken from cross-validation, whereas NLDAS-2 and PRISM predictions come from the complete models. The DVs are drawn from all MADIS observations included in XIS-Humidity, except for a small number of observations for which NLDAS-2 or PRISM is missing values.

Table 6 shows the results. While all three models improve substantially upon the SD for both DVs, the differences between the models can themselves be substantial. XIS shows improvements over PRISM which grow over time, which for humidity go from 0.10 K in 2003 to 0.33 K in 2023. The greater advantage of XIS in later years is likely due to the growth of MADIS. PRISM, in turn, is about half a kelvin better than NLDAS-2. For early years of heat index, PRISM outperforms XIS.

**Table 6.** SDs and RMSEs (K) from a comparison of two third-party models (NLDAS-2 and PRISM) with XIS. These statistics are weighted, with the weights recomputed with the reduced dataset from the main CV.

Year	Observations	Sites	Humidity				Heat index			
			SD	NLDAS	PRISM	XIS	SD	NLDAS	PRISM	XIS
2003	643,916	3,525	10.67	2.68	1.99	1.88	11.04	2.20	1.61	2.01
2004	885,577	4,430	10.47	2.56	1.90	1.76	10.66	2.14	1.53	1.79
2005	1,331,034	6,368	10.46	2.53	1.83	1.69	11.03	2.22	1.54	1.66
2006	1,789,646	7,692	10.40	2.68	1.96	1.79	10.62	2.21	1.56	1.56
2007	2,107,591	8,748	10.94	2.67	1.99	1.74	11.34	2.24	1.60	1.55
2008	2,240,395	9,644	10.86	2.47	1.86	1.45	11.20	2.13	1.60	1.36
2009	2,431,490	9,748	10.80	2.57	1.76	1.46	11.13	2.30	1.53	1.38
2010	2,626,331	10,586	10.67	2.32	1.71	1.40	11.47	2.16	1.51	1.35
2011	2,938,942	11,724	10.78	2.46	1.80	1.41	11.61	2.22	1.56	1.29
2012	3,413,705	12,828	10.38	2.51	1.82	1.37	10.68	2.18	1.55	1.22
2013	3,633,062	13,819	11.09	2.42	1.78	1.37	11.48	2.25	1.56	1.23
2014	3,864,521	14,199	11.21	2.49	1.85	1.37	11.40	2.28	1.59	1.20
2015	3,986,250	14,744	10.66	2.23	1.75	1.28	11.02	2.21	1.53	1.21
2016	3,987,549	14,895	10.18	2.16	1.68	1.28	10.53	2.21	1.53	1.25
2017	3,942,411	13,987	10.59	2.27	1.76	1.26	10.95	2.29	1.57	1.22
2018	3,949,771	14,195	11.23	2.29	1.76	1.29	11.58	2.29	1.56	1.16
2019	4,691,115	19,148	11.01	2.30	1.78	1.27	11.69	2.25	1.54	1.15
2020	5,308,228	19,190	10.57	2.30	1.75	1.28	10.93	2.22	1.55	1.14
2021	5,748,972	20,530	10.95	2.33	1.67	1.29	11.11	2.22	1.49	1.14
2022	6,166,279	21,405	11.45	2.48	1.86	1.43	11.97	2.33	1.58	1.16
2023	6,439,589	22,574	10.33	2.40	1.60	1.26	11.06	2.47	1.44	1.16

### 3.2. New predictions

**Figure 5.**
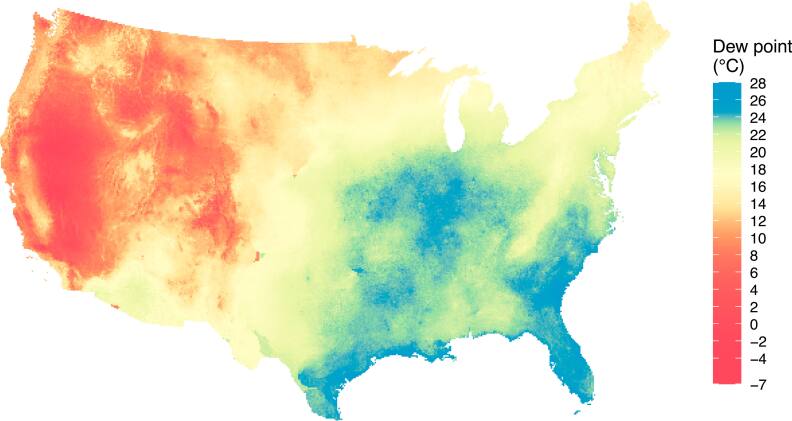
Predicted dew point for 28 Jul 2010 across the study area, shown in the US National Atlas projection. We chose this date for having the highest mean dew point in 2010 across all stations. The underlying prediction grid has cells about 9,097 m apart.

**Figure 6.**
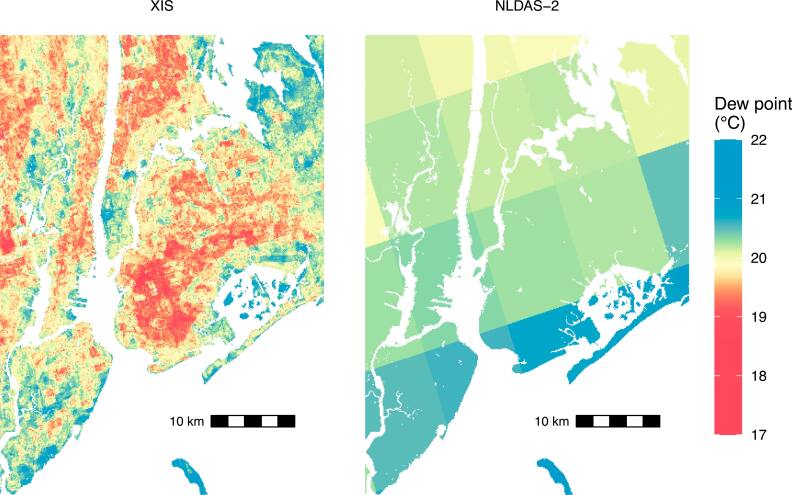
Predicted dew point for 28 Jul 2010 in the New York City area, comparing XIS to NLDAS-2. Areas of water have been masked out of the plot. In the center is Brooklyn; Staten Island lies to the southwest, Manhattan to the northwest, and Queens to the north. The underlying prediction grid has cells about 110 m apart.

For the following maps and analysis, we fit XIS to all the training data we had for each year and made predictions for new point-days. Figure 5 maps predictions for the entire study area on the most humid day in 2010. Figure 6 shows predictions for the same day in the New York City area and shows NLDAS-2’s predictions for comparison.

#### 3.2.1. Model application to social vulnerability

Previously [14], for an example application of XIS-Temperature to health, we examined the relationship of temperature and the social vulnerability index [29] in 2010. Here we extend this result to heat index, using NLDAS-2 and PRISM as comparison models.

Covering 2010-06-01 through 2010-08-31, we fit a mixed-effects linear regression model where the unit of analysis is the 71,712 US Census tracts in our study area, and the dependent variable is the mean of the mean heat index at the center of population of each tract. The model has a fixed intercept, a fixed effect for vulnerability, per-county random slopes of vulnerability, and per-county random intercepts (with the slopes and intercepts modeled as correlated). The fixed effect of vulnerability is estimated as 0.75 K ([0.71, 0.80]), where the latter is a 95% confidence interval, meaning that a change from minimum to maximum vulnerability is associated with a 0.75 K higher mean heat index over this summer.

Similar mixed models with heat-index estimates from the other models show substantially smaller estimates for this effect: 0.26 K ([0.21, 0.31]) for NLDAS-2 and 0.27 K ([0.23, 0.31]) for PRISM.

## 4. Discussion

Like XIS-Temperature, XIS-Humidity varies in accuracy over time, space, and the three DVs. As always, extrema in unusual conditions are more difficult to estimate than central tendencies in typical conditions. Overall, however, XIS-Humidity is accurate, keeping RMSEs below 3 K. XIS also beats out two competing models in all years of XIS’s timespan while XIS-derived heat index wins out in all but the first three years. An analysis of social vulnerability shows that this difference in accuracy can translate to substantial differences in the estimated relationship between heat index and social conditions. That is, XIS, compared to NLDAS-2 or PRISM, reveals greater disparities in exposure to summer heat, a finding that might motivate greater investment in adaptive capacity among those experiencing heightened exposures. XIS improves upon both plain IDW and linear regression with the same predictors, the most important predictors being the IDW values, humidity from ERA5-Land, spatial positioning, distance from water, and population density.

Modeling temperature and humidity separately, each with three DVs, provides flexibility. A common framework for temperature and humidity avoids the error that could arise from, for example, computing heat index using maximum temperature and mean humidity. Equipped with XIS, a health researcher can not only compute various widely used heat indices, but also examine separate effects of temperature and humidity, and try out new biometeorological indices to combine them. Potential public-health applications of humidity and heat-index reconstructions include (a) exposure modeling, epidemiological analysis, and health-impact assessment of heat stress; (b) studies of humidity-related health effects, such as effects on respiratory diseases; and (c) studies of ecological processes, such as those affecting vector-borne disease risks. The need for better research in these areas is growing in a warming climate. There is also growing need for non-health applications of humidity models, as in studies of energy demand for cooling, or studies of economic effects on agriculture and forestry.

Examining how predictors relate to predictions, we see that ERA5’s humidity product is more important to XIS for more isolated stations. This makes sense because the IDW predictor is less informative when other stations are farther away. Our findings for distance from water are more surprising: a positive slope for a substantial portion of the predictor’s range shows that in some conditions, the model associates greater humidity with greater distance from water. Still, overall, predictions of humidity are greater at points closer to water. This speaks to a limitation of XIS-Humidity that arises from its heavy dependence on IDW and ERA5 humidity: all other predictors are used mostly to explain leftover error after these more important predictors are accounted for. Much of the true effect of distance from water is already incorporated in IDW and ERA5 humidity, and thus won’t appear in the SHAP of distance from water.

As an interpolative statistical model, XIS is limited in both time and space. Many of its ideas could be used for modeling outside the contiguous US, but not all its predictors are available everywhere: in particular, many parts of the world have no observation network comparable to MADIS. Forecasting, or performing what-if analyses such as estimating the effects of changed land cover, are practically out of the question, because XIS depends heavily on IDW and ERA5 humidity. Even within the space and time XIS covers, we should expect lower accuracy in very sparsely observed areas, because the IDW predictor will be less informative. XIS should also be less accurate on coasts, where query points can’t be fully surrounded by observations for use with IDW, and ERA5-Land is often missing and must be replaced with plain ERA5. Having a coarser spatial resolution than ERA5-Land, plain ERA5 may produce less precise predictions from XIS. When using XIS as a predictor in epidemiological analyses, we would expect that non-differential measurement error in the exposure would typically bias observed effect estimates towards the null, with more potential for this attenuation in the earliest years of XIS with the greatest error.

A national model, based on a national dataset (MADIS), may be suboptimal for any one small area of the US. Another limitation we inherit from MADIS is the relative paucity of data in early years. The number of observations and stations in XIS-Humidity increases about tenfold from 2003 to 2024. This may explain XIS’s inferiority to PRISM in predicting the heat index for some early years. XIS’s beginning year is much later than that of NLDAS-2 (1979) and PRISM (1981, for daily estimates), and excludes it as an option for studies of 20th-century health outcomes. Overall, for the study of dew point throughout the US in the years XIS covers, we recommend XIS; for heat index before 2006, we recommend PRISM; and for highly local studies, we suggest suitably local models when available, presuming they can beat XIS’s accuracy.

An area of future work in our lab is the quantification of uncertainty. XIS produces only point predictions, without a per-prediction estimate of error. The RMSEs from the CV reported in this paper are easy to understand and likely accurate, but fall short of the kind of per-prediction uncertainty estimates that could be used for propagating uncertainty into epidemiological models. Such estimates could be produced with resampling methods, an additional model, or extensions to XGBoost itself.

## Data Availability

The data that support the findings of this study are openly available at the following URL/DOI: https://zenodo.org/doi/10.5281/zenodo.7331250 [30].
